# Prediction of U.S. daily mask wearing and social distancing using psychologically valid agents during three waves of COVID-19

**DOI:** 10.3389/fepid.2025.1532553

**Published:** 2025-05-14

**Authors:** Choh Man Teng, Peter Pirolli, Archna Bhatia, Kathleen Carley, Bonnie Dorr, Christian Lebiere, Brodie Mather, Konstantinos Mitsopoulos, Don Morrison, Mark Orr, Tomek Strzalkowski

**Affiliations:** ^1^ Florida Institute for Human and Machine Cognition, Pensacola, FL, United States; ^2^ School of Computer Science, Carnegie Mellon University, Pittsburgh, PA, United States; ^3^ University of Florida, Gainesville, FL, United States; ^4^ Department of Psychology, Carnegie Mellon University, Pittsburgh, PA, United States; ^5^ Department of Cognitive Science, Rensselaer Polytechnic Institute, Troy, NY, United States

**Keywords:** cognitive model, COVID-19, decision making, behavior, ACT-R

## Abstract

We present Regional Psychologically Valid Agents (R-PVAs) as a modeling approach to predicting transmission-reducing behaviors and epidemiology. The approach builds upon computational cognitive theory and formalizes aspects of theories of individual-level behavior change. We present R-PVA models of social distancing and mask wearing in response to dynamics in the physical and information environments in the 50 U.S. states. The models achieve strong goodness-of-fits for predicting day-to-day mask-wearing (*R*^2^ = 0.93) and social distancing (*R*^2^ = 0.62) for the first three waves of COVID-19, prior to the rollout of vaccines.

## 1 Introduction

Human behavior plays a crucial role in controlling the spread of infectious diseases, whether in the form of pharmaceutical (e.g., vaccines) or non-pharmaceutical interventions (e.g., wearing masks; social distancing) (1). Epidemiological models often include stylized abstractions of human behavior and interaction (2–6), but do not include theoretically established, empirically validated, computational models of human psychology and social processes. Since the onset of the COVID-19 pandemic we have focused on the development of Psychologically Valid Agents (PVAs) based on established computational cognitive theory (7–12). We propose that richer, more accurate models of human psychology and behavior can improve epidemiological modeling and public health decisions (8).

**Figure 1:**
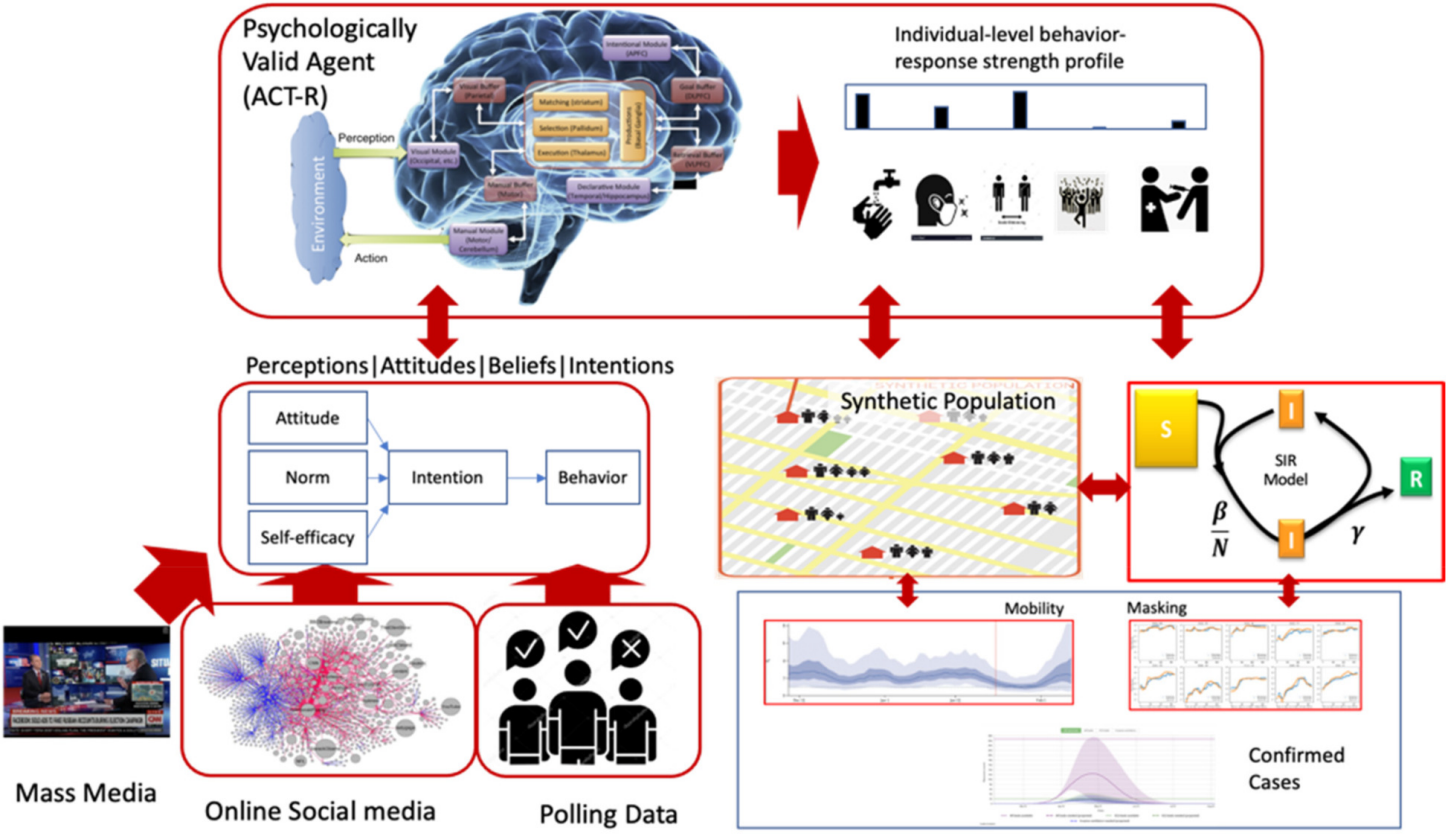
The PVA framework. Clockwise from lower left: Datasets are used to extract cognitive content (e.g., perceptions, attitudes, beliefs, and intentions) and initialize PVAs implemented in the ACT-R cognitive architecture. Given decision contexts, the PVAs simulate the choice of behaviors, which predict behavior-response profiles (e.g., for hand washing, social distancing, vaccination). The PVAs can be run in agent-based models or abstracted into SIR models to simulate effects on disease progression.

The PVA approach is presented in Figure 1. The goal is to model beliefs, attitudes, intentions, and behavior that are assumed to influence the transmission of infectious diseases. The core cognitive modeling is based on the ACT-R cognitive architecture (13). The model relies on analyses, including natural language processing and machine learning techniques, that process a mix of big data sources including online media such as Twitter and mass media. PVAs are used to model behavior change, such as social distancing and mask wearing in response to dynamics in the physical and information environments and are designed to represent individuals or homogeneous segments of a population of interest. In recent work (12) we presented Regional PVA (R-PVA) models of mask-wearing behavior in the 50 U.S. states. Here we present an extension of those R-PVAs to include mass media and social media analyses and surveyed perceptions of local infections. The extended R-PVAs are used to predict social distancing behavior in addition to mask-wearing.

## 2 ACT-R and R-PVA models

Leveraging extensive COVID-19 data repositories and other general data sources, we created Regional PVAs to simulate the behavior of regional U.S. populations during the pre-vaccination phase of COVID-19. The abundance of regionally organized (e.g., state, county) demographic, psychographic, epidemiological, behavioral, and information environment data makes it feasible to develop and test such models. The PVA pipeline includes demographic and psychographic data about U.S. regions and online social media. These data are used to initialize agents and provide time-series inputs representing the pandemic context (e.g., local transmission rates). The PVAs iteratively assess the current context and make decisions (e.g., wear a mask or not) over discrete time steps (e.g., every day). They are thus capable of predicting various regional timeseries data, e.g., the U.S. county- or state-level daily mobility patterns or daily mask-wearing. Regional PVAs rely on ACT-R Instance-based Learning mechanisms to conduct online learning, continuously adapting to daily data inputs. Regional PVAs can be used as a novel data mining technique to understand possibly nonlinear relations between context and behavior.

In previous work (12), R-PVAs were developed to model mask-wearing behavior in the U.S. over the pre-vaccination phase of COVID-19 using regionally organized demographic, psychographic, epidemiological, information diet, and behavioral data. We also modeled a process of gaining self-efficacy with repeated behavior. Self-efficacy is a theoretical mechanism in social psychology: An individual's belief in their capacity to execute behaviors necessary to produce specific performance attainments. The Social Cognitive Theory of self-efficacy (14) predicts that behavioral goals that are perceived as too difficult are unlikely to be attempted. In general, greater levels of self-efficacy lead to greater likelihoods of achieving a goal. An R-PVA using a set of five regional predictors selected by stepwise regression, a psychological self-efficacy process, and context-awareness of the effective transmission number, *R_t_*, yielded good fits to the observed proportion of the population wearing masks in the 50 U.S. states [*R*^2^ = 0.92]. An R-PVA based on regional Big 5 personality traits yielded strong fits [*R*^2^ = 0.83]. R-PVAs are a novel technique to understand dynamical, nonlinear relations amongst context, traits, states, and behavior based on cognitive modeling.

## 3 Modelling pipeline

**Figure 2:**
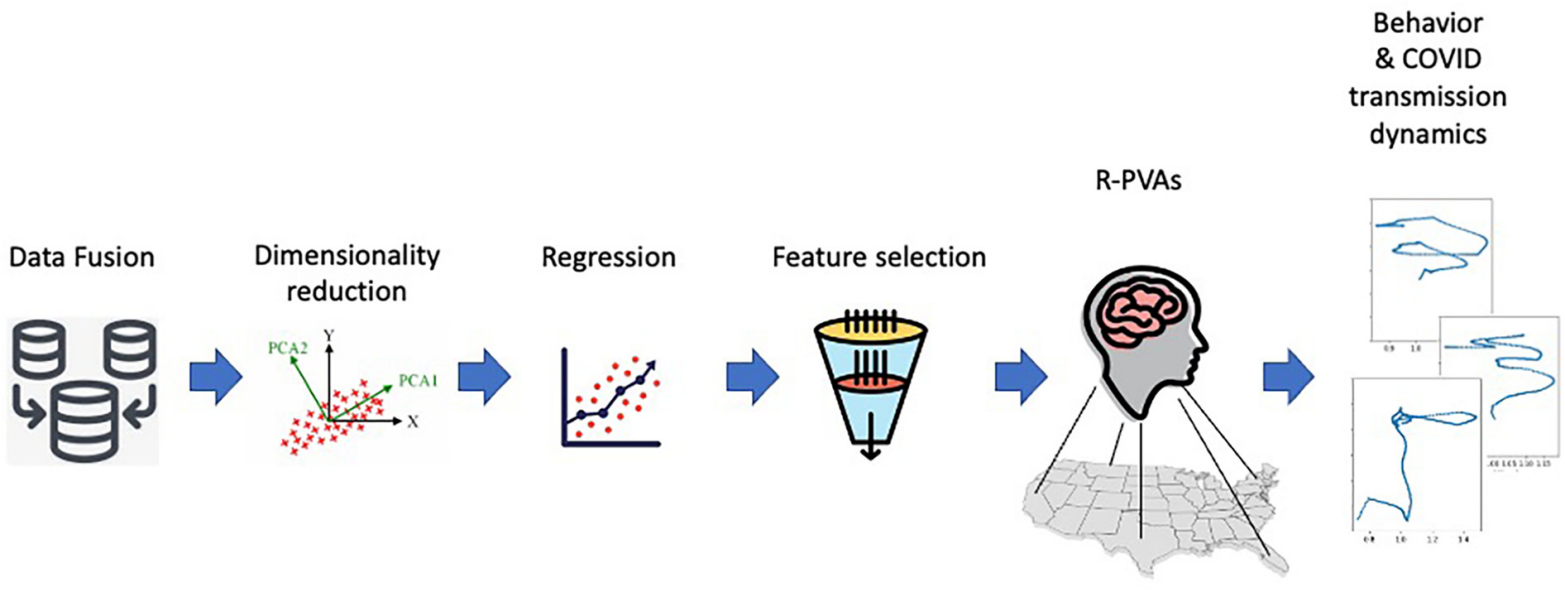
The modelling pipeline.

The modelling pipeline is summarized in Figure 2. Data from a variety of sources were collected and preprocessed as described in Section 4. The merged data set consists of (1) static variables describing regional characteristics such as demographics and political leanings, (2) time series variables describing dynamics such as epidemiological phenomena as well as stances and topic mentions in mass media and social media, and (3) behavioral variables for the adherence to the non-pharmaceutical interventions of mask wearing and social distancing. The standardized sets of variables (1) and (2) were used to predict the behavioral choices (3) that could have an impact on the spread of infectious diseases.

Some variables may have a longer temporal range effect on other variables. For instance, *R_t_* is not directly observable and the effect of a change in *R_t_* would manifest itself only after an incubation period. We performed a Granger analysis to identify the time lags between variables that would produce a significant effect. The resulting shifted time series together with the static predictor variables were downselected using stepwise regression. The most significant variables were then used to construct the R-PVA models. In addition, modelling the self-efficacy mechanism involves two parameters. A grid search was performed on a small data set to estimate the optimal parameter values.

We modelled the behavioral variables using R-PVAs constructed with variable sets of several sizes, with and without additionally modelling the effect of the self-efficacy mechanism. The evaluation consisted of cross-validation using unseen data from successive time periods. We will describe some of the analysis steps in more detail in the following sections.

## 4 Data and feature construction

In previous work we modelled mask wearing behavior in the United States using demographic, epidemiological, psychographic, weather, political and media diet data at the state and county levels. Here we expanded the data set to include additional inputs, including COVID- and influenza-like illnesses, pro/con stances and other measures derived from social media, and topic mentions in mass media. We validated the model on a social distancing measure in addition to mask wearing. Geographic location information was not used as an input for the cognitive model, but regional differences were observed from the modelling analysis.

Supplementary Tables S1, S2 in Supplementary Materials show the static and time-series data used in this study. Using principal component analysis, the raw monthly weather data was reduced from 48 dimensions to 2 dimensions. The first principal component (PC1) explains 65% of the variance and is dominated by the monthly temperature related variables. The second principal component (PC2) explains 13% of the variance and is dominated by the monthly precipitation related variables during the fall and winter (Oct–Apr).

The Stanford Cable TV News Analyzer made available closed caption transcripts time-aligned with the video recordings of TV news programming on CNN, Fox News, and MSNBC. We compiled co-mentions of terms for “COVID”, “masks” and several attitudinal concepts, such as “protect”, “restrict”, “fear”, as well as general positive and negative descriptions (e.g., “good”, “useless”). Supplementary Section S1 in Supplementary Materials describes the procedure for obtaining the mass media data in more detail.

Except for the social media data from CASOS, which has a shorter time range (04/08/2020–11/30/2020), the time series data covers the pre-vaccination rollout period of 04/24/2020–03/31/2021, corresponding to the first three waves of COVID-19 as defined by Pew Foundation^1^. Most of the data is daily and available at both the state and county levels. A notable exception is the mask wearing data. COVID States data was collected approximately every three weeks, starting in April 2020. COVIDcast data was collected daily but with a later start date of September 2020. We imputed the time series by first ordering the survey data by the date that occurred mid-survey wave and iterating through each successive pairs of data points using linear interpolation weighted by the ratio between elapsed days from the lower-dated value and days remaining to the upper-dated value.

### 4.1 Exploratory analysis

We performed pairwise correlation between the variables (see Supplementary Figure S1 in Supplementary Materials). Each variable consists of *nt* data points, where *n* is the number of states and *t* is the number of days in the target period. The two target behavioral variables, mask wearing and difference in daily social distance, were moderately negatively correlated (*r* = −0.41). Larger increases in the proportion of mask wearing were associated with larger decreases in distance travelled compared to the pre-COVID baseline. Both target variables were most highly correlated with the political variables (*r* = ±0.41–±0.53), which were themselves very highly correlated (*r* = 0.77–0.89). The political variables however were not very correlated with any of the mass media variables, for example, the proportion difference in people watching Fox News (*r* = −0.02–0.07). CDC case rate was moderately correlated with COVID- and influenza-like illnesses (*r* = 0.35–0.40), but not much correlated with *R_t_* (*r* = −0.06). The latter might be an indication of the time lag between *R_t_* and the onset of observable symptoms. We will re-align the data to account for this effect as identified by Granger analysis.

### 4.2 Granger analysis

As mentioned, some variables might not have an immediate effect on others. We performed Granger analysis on pairs of time series variables. For each pair of variables *x* and *y*, the predictor variable *x* was shifted with successive time lags of 1–30 days, and the shifted previous values of *x*, together with the unshifted previous values of the predicted variable *y*, were used as input to predict the current value of *y*. The shortest time lag of *x* with a significant improvement over using only the values of *y* was deemed the effective lag length. Bonferroni correction to the level of significance (0.05) was applied to tests of each *x-y* pair.

**Figure 3:**
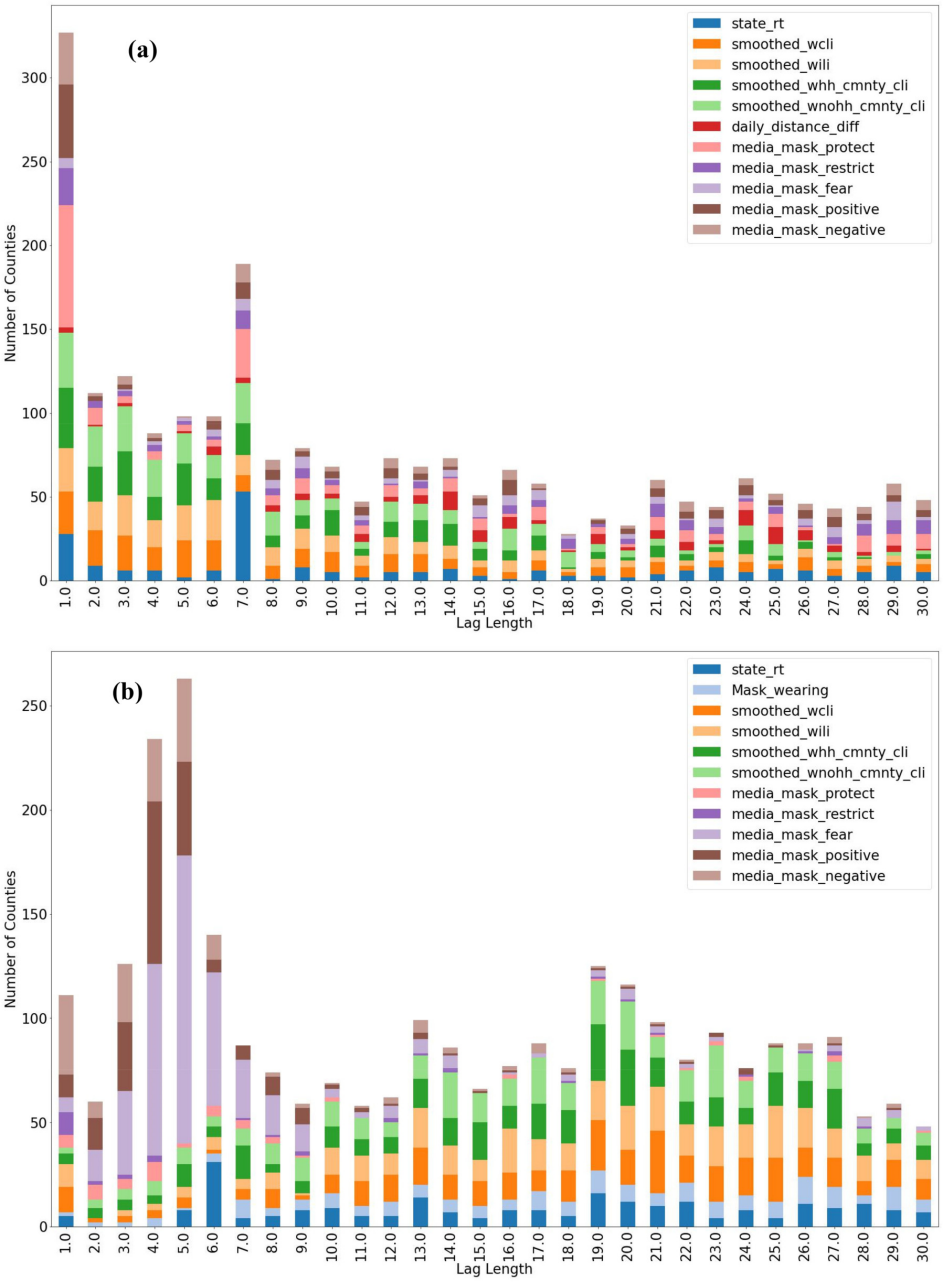
Granger analysis results for **(a)** mask wearing and **(b)** social distancing. Time series variables are described in the Supplementary Materials.

Figure 3 shows the time lags of variables predicting the proportion of mask wearing and the difference in daily distancing at the county level, which provide a finer-grain view of the relationships than at the state level. For mask wearing, the most common lag length for *R_t_* is 7 days, which could be partly attributed to the incubation period of COVID-19. Other more observable epidemiological indicators, such as COVID-like and influenza-like illnesses had a slightly shorter time lag. For daily distancing difference, mass media indicators had a wider influence in the shorter time frame, whereas *R_t_* and the illness indicators had more uniform influence over the longer time lags. Note that not all time series variables in the data set appeared in the figure, because some of them, notably the social media variables, did not exhibit a significant lag with respect to the target variables (mask wearing and social distancing) according to Granger analysis.

Where applicable the variables were shifted by their respective effective lag lengths to account for the time lags between the variable and the behavior variable of interest. All subsequent analyses were performed using the time-shifted data.

### 4.3 Feature selection

The most relevant features were selected using forward stepwise regression on the shifted data. Starting with an empty set of predictors, at each step a linear regression model for predicting the target variable was constructed using the predictors selected so far and one of the unselected predictors. The models were evaluated using 10-fold cross validation, where the data set was randomly divided into 10 folds, and for each trial 9 folds were used for training and the remaining 1 fold was used for testing. The unselected variable that gave rise to the model with the highest average *R^2^* was added to the set of selected variables.

**Figure 4:**
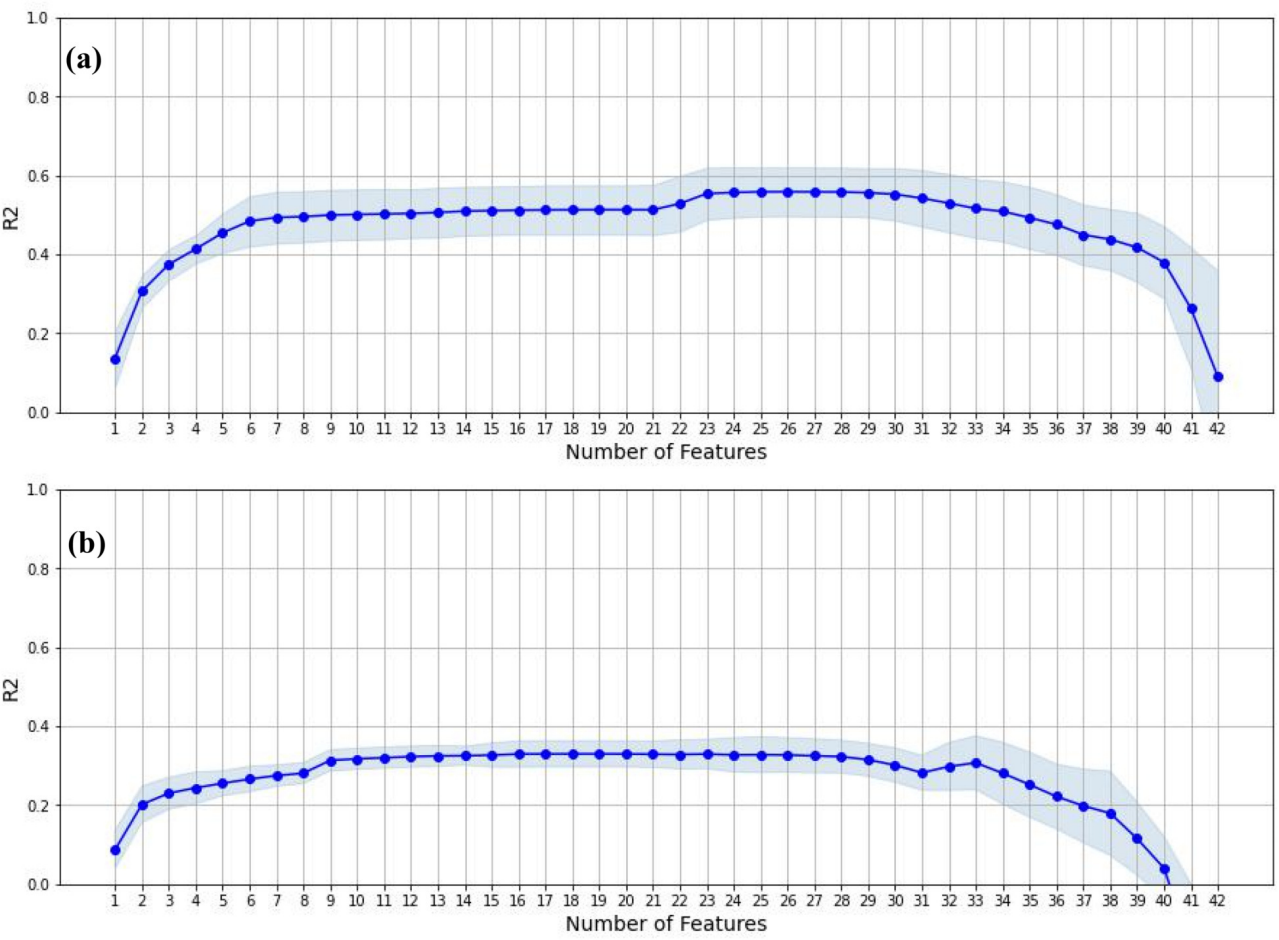
Overall goodness-of-fit for feature selection in stepwise regression for predicting **(a)** mask wearing and **(b)** social distancing.

Figure 4 shows the *R^2^* of successive models with increasing number of predictors selected. The optimal cutoff was obtained by visually inspecting the graph and finding the “shoulder” where adding more variables did not result in much more improvement in the *R^2^* score. For mask wearing, the optimal cutoff was set at 6 variables. For daily distancing difference, the optimal cutoff was less clear. We selected two potential cutoff points, at 3 and 9 variables, for further exploration. The variables selected in each case are shown in Table 1.

**Table 1:** Best predictors for mask wearing and social distancing.

Mask Wearing	Daily Distance Difference	
	(3 variables)	(9 variables)
PctTrump_State_2016	trump_approval_feb2020	trump_approval_feb2020
Agreeableness	smoothed_wcli	smoothed_wcli
smoothed_whh_cmnty_cli	Population	Population
Openness		media_mask_restrict
media_foxnews_lean		Median_Household_Income_2018
TOM		smoothed_wili
		Total_age65plus
		smoothed_whh_cmnty_cli
		smoothed_wnohh_cmnty_cli

For both mask wearing and daily distance difference, the predictor with most weight was a variable representing the political leaning towards Trump. For mask wearing, the additional variables spanned a variety of dimensions, including personality, epidemiology, media and demographics. For daily distance difference, the 3-variable set included additionally COVID-like illness and Population, whereas the 9-variable set was more heavily focused on the COVID-like and influenza-like illness indicators. As shown in Supplementary Figure S1 in the Supplementary Materials, these indicators were highly correlated. From Figure 4b we observed that the additional 6 variables gave rise to a noticeable though modest increase in *R^2^* over the 3-variable model. Overall, the best *R^2^* achieved by the mask wearing models were considerably larger than that of the daily distance models, indicating that the mask wearing model was a better fit than the daily distance model.

## 5 Regional psychologically valid agents

Regional Psychologically Valid Agents can be viewed as a variation on recent research on geographical psychology. This is a rapidly growing subfield that has generated a 588% increase in publications between 2001 and 2019 (15). Geographical psychology studies the spatial patterns of psychological phenomena, including the spatial distribution of psychological traits, underlying mechanisms shaping psychological patterns, and their influence on micro- and macro-level patterns. For instance, geographical psychology studies (e.g., 15, 16) show spatial clustering of psychological characteristics and psychological phenomena associated with economic entrepreneurship and other political, economic, social, and health outcomes.

### 5.1 ACT-R and R-PVA models

Our R-PVAs utilize a decision-making modeling approach known as Instance-Based Learning (IBL) (17, 18). Such models are particularly effective in dynamic and uncertain environments. The R-PVA models implementing IBL were developed in pyACTUP 2.2.3, which simulates the declarative memory module of ACT-R, including the storage and retrieval of experiences and a memory blending process. IBL leverages the ACT-R framework to simulate human decision-making based on past experiences stored in declarative memory. This memory stores multi-attribute past instances of decisions with subsets of attributes representing *contexts* (situational features), *actions* (decision; behavior choice), *outcomes* (resulting changes), and *rewards.* When a decision needs to be made, IBL uses a memory retrieval process called *blended retrieval* (or blending) to compute a choice or behavior based on decision context. Rather than retrieving a single best-matching past memory instance, blending computes a weighted average of multiple stored memory instances. The contribution of each instance to the blended result is determined by the *activation*, which is based on frequency and recency of the instance, and the *similarity* of the instance contexts to the decision context. Blending makes context-sensitive decisions that integrate past experiences and allows for generalization and adaptation in dynamic environments.

The mathematical foundation of IBL is rooted in Statistical Learning Theory (19), where it functions as a linear smoother—a non-parametric, instance-based learning function approximator. Consequently, IBL supports various learning paradigms, including Supervised Learning for regression and classification, as well as Reinforcement Learning for utility-based habitual behaviors. IBL continuously retains its “training data” within its memory repository, allowing it to adapt dynamically to new situations. To handle large datasets efficiently, IBL computations can be vectorized and parallelized, incorporating techniques such as approximate k-nearest neighbors for scalability. This combination of scalability, adaptability, and a cognitively plausible approach to decision-making through approximate expectations makes IBL particularly well-suited for modeling evolving human behavior in large-scale multi-agent systems.

ACT-R has subsymbolic mechanisms that determine the dynamics of the R-PVAs. Equations 1–3, below, define how the levels of activation of chunks in memory determine the probabilities of chunk retrieval.

Blended retrieval determines the value *V* that minimizes the sum of squared dissimilarities with the answer *V_i_* proposed by each chunk, weighted by the probability, *P_i_*, of retrieval of value *V_i_*:

(1) $$V=\underset{V}{\mathrm{argmin}}\;\;\sum_{i}P_{i}{\left(1-Sim(V,V_{i})\right)}^{2}$$

The probability of retrieval is

(2) $$P_{i}=\;\frac{e^{A_{i}/s}}{\sum_{j}\;e^{A_{j}/s}}$$

Where the activation, *A*_*i*_, is

(3) $$A_{i}=B_{i}+\sum_{f}MP_{f}\;\mathrm{Sim}(\;f,V)+\;\varepsilon_{i}$$

and *s* and *ɛ* are noise factors, *B* is a base-level activation, and MP. MP*_f_* is a mismatch penalty representing the dissimilarity between the representation of two values. Equation 4 defines how activation levels are increased by repeated experiences, or decay with time.

(4) $$B_{i}=\ln\;\left(\sum_{\;j=1}^{n}t_{j}^{-d}\right)+\beta_{i}$$

where *t_j_* is the time since the *j^th^* storage or retrieval trial of chunk *i*, *n* is the number of occurrences, 0 < *d* < 1 is a decay parameter, and *β*_*i*_ is a constant offset. The parameters were set as follows: *MP* = 30.0, *d* = 0.5, and *β* = 0.0. The mismatch penalty *MP* was set to a value that allowed a relatively broad range of inexact matches to contribute to the activation level, weighted by the magnitude of the mismatch. The decay parameter *d* was set to the mid-point of the range, allowing for a moderate rate of decay.

Chunks generally can be represented as an unordered feature-value list of the form

{<feature_1_: value_1_>, <feature_2_: value_2_>, …, <feature_n_: value_n_>}

For the R-PVAs modelling a target predicted behavior, the chunks are of the form

{<predictor_1_: value_1_>, …, <predictor_n_: value_n_>, <predicted: value_p_>}.

For instance, for modelling mask wearing, if on a given day *t* a given state had a mask wearing proportion of *x* and a value of *v_t_* for the time series variable smoothed_whh_cmnty_cli, then we learned a total of 10 new chunks of two forms proportional to *x*, that is, round(10*x*) chunks of the form

{<smoothed_whh_cmnty_cli: v_t_>, <PctTrump_State_2016: v_1_>,…, <TOM: v_6_>, <mask_wearing: 1>}

corresponding to the mask-wearing faction, and 10-round(10*x*) chunks of the form

{<smoothed_whh_cmnty_cli: v_t_>, <PctTrump_State_2016: v_1_>,…, <TOM: v_6_>, <mask_wearing: 0>}

corresponding to the non-mask-wearing faction. These chunks together with the previously learned chunks formed the current memory of the model.

To make a prediction of the prototypical value of a feature given a (partial) set of predictor values, chunks that are similar to this chunk are retrieved and blended, weighted by a similarity function. Our R-PVA models used the similarity function

(5) $$Sim(x,y)=1/(1+\exp\;(y-x){)}^{2},\mathrm{with}\;y>x.$$

Thus, the mask wearing proportion of the next day, *t* + 1, was obtained by the blended retrieval of mask_wearing, given the values of the predictor variables on day *t* + 1:

{<smoothed_whh_cmnty_cli: v_t_ _+_ _1_>, <PctTrump_State_2016: v_1_>,…, <TOM: v_6_>},

using the smoothed_whh_cmnty_cli value of day *t* + 1, and the same values for the static variables as before. (Recall that the values of the static variables varied from state to state, but for a given state, their values remained constant from day to day.) Once the mask wearing proportion for day *t* + 1 was obtained, another 10 new chunks for *t* + 1 corresponding to this proportion were generated and incorporated into the model. This learning-prediction process was repeated for each day forward.

### 5.2 Self-Efficacy

In general, behavior change involves the gradual development of habits that are woven into the fabric of everyday life (20). A long history of psychological and neuroscience research on learning, decision-making, and behavior has led to the idea that there is a dichotomy between decisions and behaviors that result from effortful goal-striving or reflective processes vs. behaviors that are more automatic, less effortful habits (21–24). The execution of novel behaviors typically involves an initial goal-striving phase that requires cognitive effort in deliberation and behavioral control in the relevant environmental contexts. Then, repeated practice produces habit formation and strengthening processes that associate those specific behaviors to cues in the environment. Habitual behaviors come to be executed without effortful goal-striving cognitive processes. Thus, generally, novel behaviors are more difficult than familiar everyday behaviors because they require goal-striving.

The construct of self-efficacy in Social Cognitive Theory is a person's judgment about their ability to control events or their own behaviors. In general, an individual's belief in their ability to perform a behavior is necessary to attain the behavior. Self-efficacy has been defined in terms of an underlying cognitive learning process that may come from experienced mastery of the behavior or vicarious observation of similar others performing the behavior (14, 25). In our models, we assume mask-wearing is for most people (e.g., those not in health care) a novel behavior that initially requires effortful goal-striving processes, and that self-efficacy processes and repeated practice gradually make the behavior more likely to be executed.

For R-PVAs that model self-efficacy, two additional features are included:

{<predictor_1_: value_1_>, …, <predictor_n_: value_n_>, <difficulty: *δ*>, <effort: *e*>, <mask_value: *m*>},

where *δ* is the difficulty of the task of mask wearing, and *e* is the amount of perceived effort to accomplish the task. Self-efficacy is modelled as the difference between the difficulty and effort. For each success in accomplishing a goal (e.g., wearing a mask for a day in our scenario), self-efficacy is boosted by

(6) (1−m)boost_factor(exp(δ−e)/(exp(δ−e)+exp(e)))

where *m* is the mask wearing proportion, boost_factor is a small quantity that promotes the self-efficacy upon success. The threshold above which the intended effort would be too hard to attempt was set at the mean of the initial difficulty and effort.

### 5.3 Norm initialization

In the current model, a “norm” is simply an initial behavioral bias in a subpopulation. During the initialization phase, a norm was established as the baseline for the model, using only the static variables and the values of the target (time series) variable from the initial 10 days of the data. For each state, the blended norm *x* of the target variable was obtained and 10 new chunks in proportion to this value *x* were learned, such that, keeping the other variable values as given, a proportion of *x* of the new chunks had a value representing the presence of the behavior (e.g., wearing a mask), and the rest of the 10 chunks had a value representing the absence of the behavior (e.g., not wearing a mask).

### 5.4 Parameter tuning

The self-efficacy mechanism in R-PVA involves two parameters, the initial effort needed to perform a behavior and the boost factor when the behavior is reinforced (without loss of generality, relative to a fixed level of difficulty and a threshold value that is a function of the other parameters). We performed a grid search over the parameter space to identify the optimal parameter setting, with the difficulty level set at 2.0 and using only data from California and Wyoming during the first wave of COVID-19 (up to 2020/06/30). The R-PVA models constructed were evaluated using *n*-fold rolling origin cross-validation (see Section 6.1).

**Figure 5:**
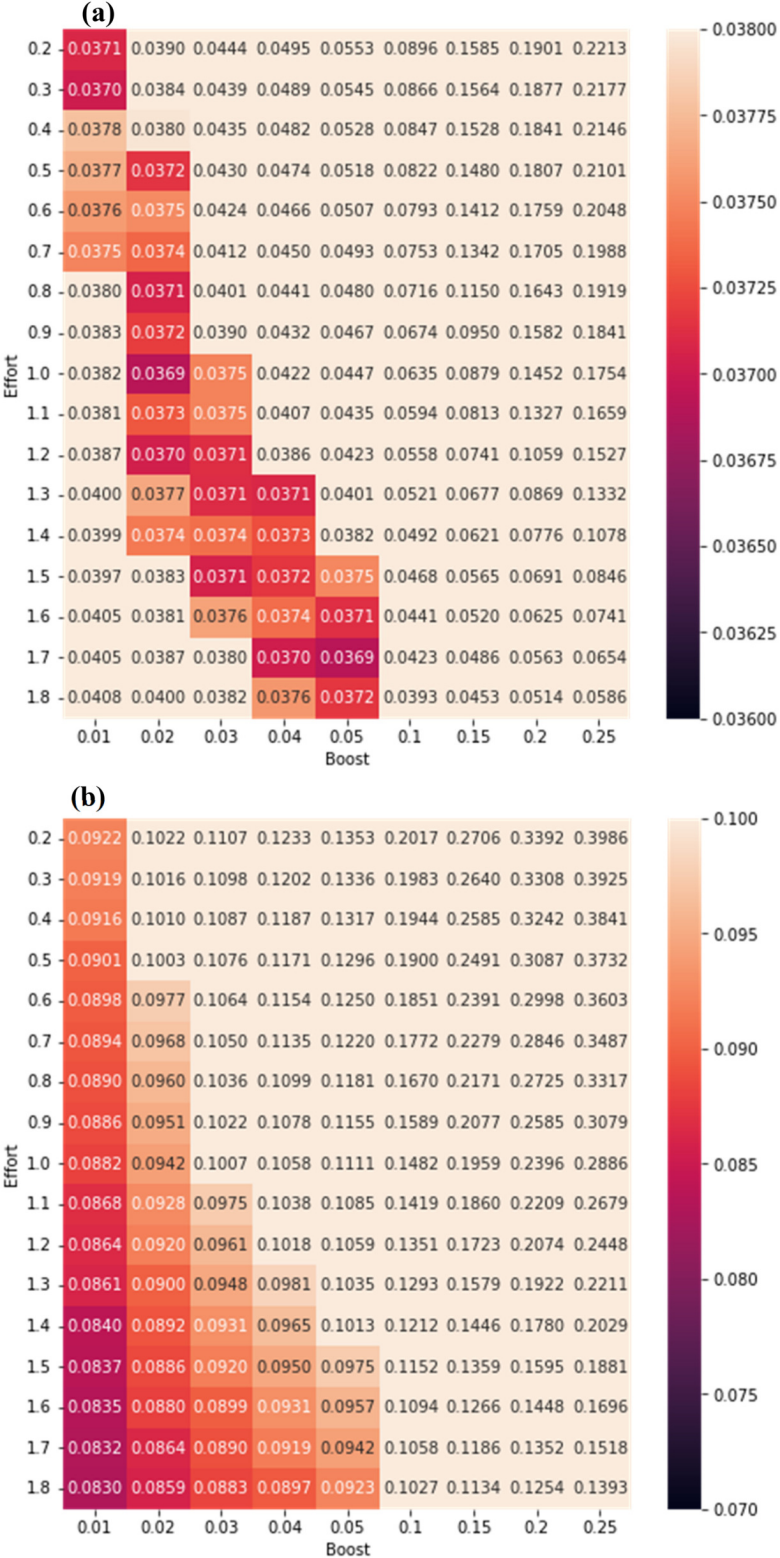
RMSE fits for parameters in predicting **(a)** mask wearing and **(b)** social distancing.

Figure 5 shows the RMSE values of models for mask wearing and daily distance difference respectively. For mask wearing, the best-performing parameter values were: boost factor = 0.02, effort = 1.0. For daily distance difference, the best-performing parameter values were: boost factor = 0.01, effort = 1.8. We noted that for daily distance difference, the optimal parameter setting was at the boundary of reasonable parameter space, which might indicate that the self-efficacy mechanism was not of high utility. We will discuss this further below.

### 5.5 Simulation of behavioral changes

Norms obtained as described in Section 5.3 provided a starting point for the models. At each time point, ten memory chunks were added in proportion to the presence or absence of the target behavior as predicted by a R-PVA model. The model then simulated the behavior for the next time step using the present external variables and a blending of the past history of the internal memory chunks up to that point, subject to a decay function. The behavioral characteristics and the new memory chunks at the next time point were then set according to the new blended value.

## 6 Evaluation and results

### 6.1 Evaluation

For each of the two behavioral variables, mask wearing and daily distance difference, we constructed R-PVA models using the variables selected from the stepwise regression procedure and the parameter values obtained from the grid search. Several models of increasing complexity were evaluated, using a subset of the selected variables:

• Model 1: only the most significant time-series variable
• Model 2: the most significant time-series variable and the most significant static variable
• Model 3: all the selected variables
• Model 4: all the selected variables, with modelling of the self-efficacy mechanism

For daily distance difference, we evaluated Models 3 and 4 with both the 3-variable and 9-variable versions of “all” variables as shown in Table 1.

Because of the sequential nature of the data, regular cross validation, with random assignment to folds, was not appropriate, as this would enable the prediction of a data point using future data points that should not have occurred yet. We instead analyze the models using *n*-fold rolling origin cross-validation (26), *n* being the length of the time series minus 1. For the *i*-th fold, the data from the initial time sequence *<t_0,_ t_1,_*…*t_i−1_>* was used for training, and the data at time point *t_i_* was used for testing. Each successive training data set was a longer time series and included the previous training set. *n*-fold rolling origin cross-validation was performed using data in the date range 2020/04/24–2021/03/31. *R^2^* and RMSE scores were obtained for each of the models.

### 6.2 Results

The evaluation results are shown in Tables 2, 3. For both mask wearing and daily distance difference, the models performed better as we included more of the selected variables (from R-PVA-*-1 to R-PVA-*-3), in particular going from the first model to the second model, suggesting that political leaning (PctTrump State 2016 and trump_approval_feb2020) had a substantial effect on the target behaviors.

**Table 2:** Evaluation of R-PVA models for predicting daily mask wearing.

Mask Wearing	Number of Predictors	Self-Efficacy	Average RMSE	Average *R^2^*
R-PVA-mask-1	1 (smoothed_whh_cmnty_cli)	no	0.10	0.22
R-PVA-mask-2	2 (+ PctTrump State 2016)	no	0.06	0.75
R-PVA-mask-3	6	no	0.04	0.86
R-PVA-mask-4	6	yes	0.03	0.93

**Table 3:** Evaluation of R-PVA models for predicting daily social distancing.

Daily Distance Difference	Number of Predictors	Self-Efficacy	Average RMSE	Average *R^2^*
R-PVA-distance-1	1 (smoothed_wcli)	no	0.13	0.20
R-PVA-distance-2	2 (+ trump_approval_feb2020)	no	0.10	0.53
R-PVA-distance-3	3	no	0.09	0.62
R-PVA-distance-4	3	yes	0.09	0.62
R-PVA-distance-3a	9	no	0.14	0.06
R-PVA-distance-4a	9	yes	0.14	0.05

The models of the two behaviors diverged with the inclusion of the self-efficacy mechanism. For mask wearing, self-efficacy helped improve the model, whereas for daily distance difference, the models with self-efficacy performed slightly worse than their corresponding models without self-efficacy [e.g., R-PVA-distance-3(a) vs. R-PVA-distance-4(a)]. We will discuss this in more detail in Section 7.

In addition, in the 9-variable setting for daily distance difference, both models without and with self-efficacy were worse than their 3-variable counterparts, suggesting that the 9-variable set might be overfitting the model. There were other indications that this might be the case. For instance, we noted earlier, when performing feature selection using stepwise regression (Figure 4b), that the addition of the 6 variables beyond the first 3 provided only a modest increase in *R^2^*. Another consideration is that perhaps daily distancing was inherently harder to predict than mask wearing, as suggested by the lower maximum *R^2^* scores achieved for the two behaviors (Figures 4a, b). Note however, that R-PVA and regression models are based on different modelling principles and the results from stepwise regression, although suggestive, are not directly extensible to R-PVAs.

We noted earlier that the variables that carried the most weight in the feature selection procedure were political variables (“PctTrump State 2016” and “trump_approval_feb2020” respectively), suggesting that political partisanship is a strong factor in the adherence to non-pharmaceutical interventions during COVID-19. Here we will delve into this aspect more closely by examining the states with the most extreme values for “PctTrump State 2016”. The five states with the highest “PctTrump State 2016” values were West Virginia, Wyoming, Oklahoma, North Dakota and Kentucky. The five states with the lowest “PctTrump State 2016” values were Maryland, New York, Hawaii, Vermont and California. We will refer to these two sets of states as *HighTrump* and *LowTrump* states respectively.

**Figure 6:**
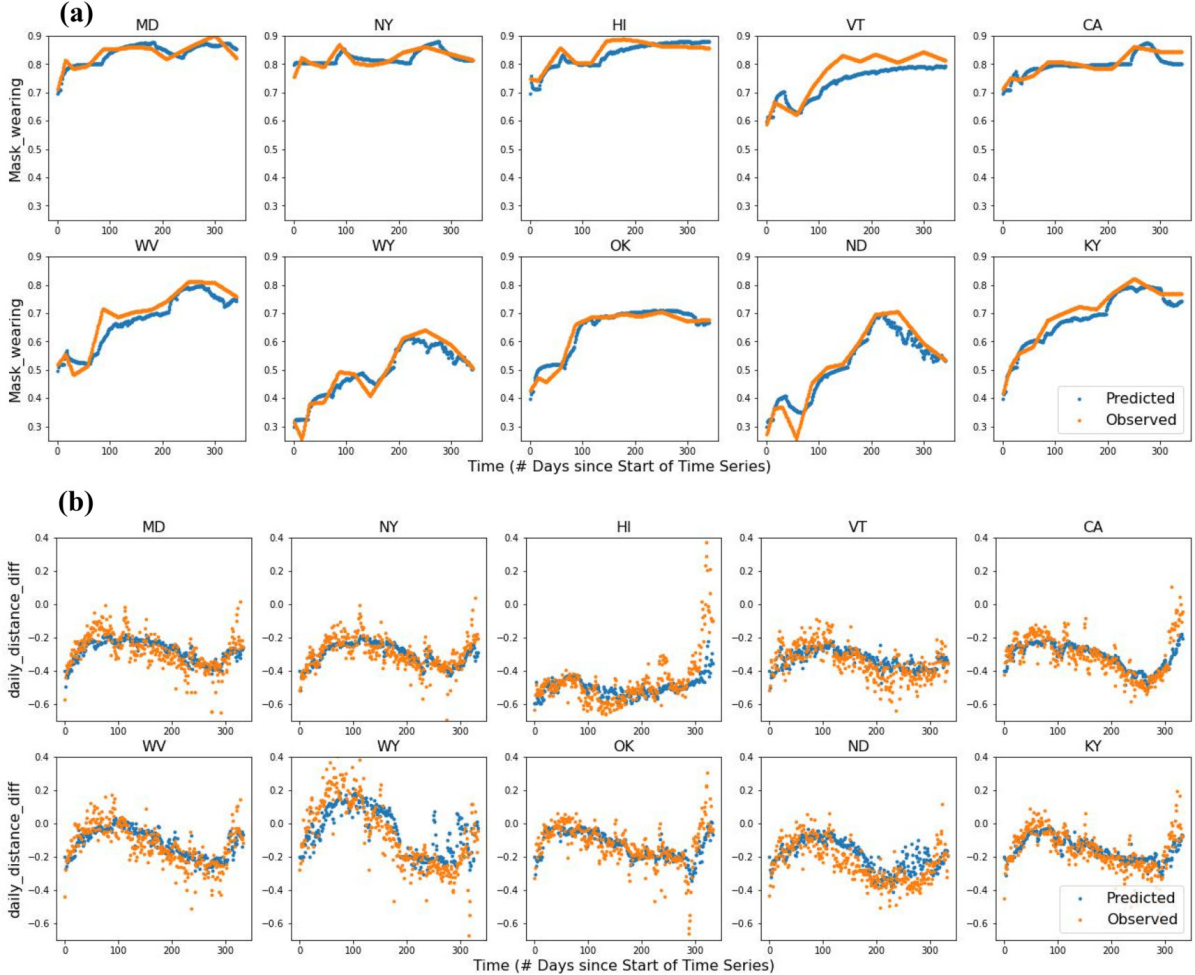
R-PVA prediction of daily behavior during COVID-19: **(a)** observed proportion of mask wearing and proportion predicted by R-PVA-mask-4, and **(b)** observed daily social distancing difference and behavior predicted by R-PVA-distance-3, for 10 U.S. states over the first three waves of COVID-19. The top row shows the LowTrump states (5 states with the lowest proportion voting for Trump in 2016) and the bottom row shows the HighTrump states (5 states with the highest proportion voting for Trump in 2016).

Figure 6 shows the observed and predicted values of the two behavioral variables in the *HighTrump* and *LowTrump* states over time. The predicted values were obtained from the best performing models of the two behaviors (R-PVA-mask-4 and R-PVA-distance-3). In general the predicted values tracked the observed values fairly closely. LowTrump states showed a higher proportion of mask wearing behavior and a larger decrease in distance travelled compared to the pre-COVID baseline. For mask wearing, the values in HighTrump states fluctuated but exhibited an overall upward trend over time, whereas the values in LowTrump states showed less fluctuation, possibly because the proportion of mask wearing was already fairly high in those states. For difference in daily distance, the values in both HighTrump and LowTrump states displayed more variations than those for mask wearing, perhaps another factor contributing to the lower performance of the social distancing models, when compared to the mask wearing models. For most states, the difference in daily distancing progressed in an S-shape, increasing steadily till around day 80 and then decreasing till around day 280 when it started to rise again, drastically in some cases. These inflection points roughly corresponded to the end of the first wave (and start of the second wave) of COVID-19 and the vaccine roll out at the end of the third wave. The HighTrump states had smaller decreases in distance travelled, but with slightly more variation than LowTrump states, and in some states there was even an increase in distance travelled compared to the pre-COVID baseline.

## 7 Discussion and conclusion

**Figure 7:**
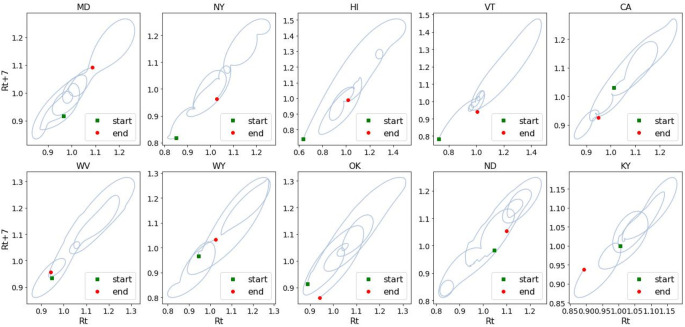
Dampened oscillation of *R_t_*. The values of *R_t_* are plotted against *R_t_* *_+_* *_7_*, where *t* is in days. Figure panel titles are U.S. state abbreviations.

**Figure 8:**
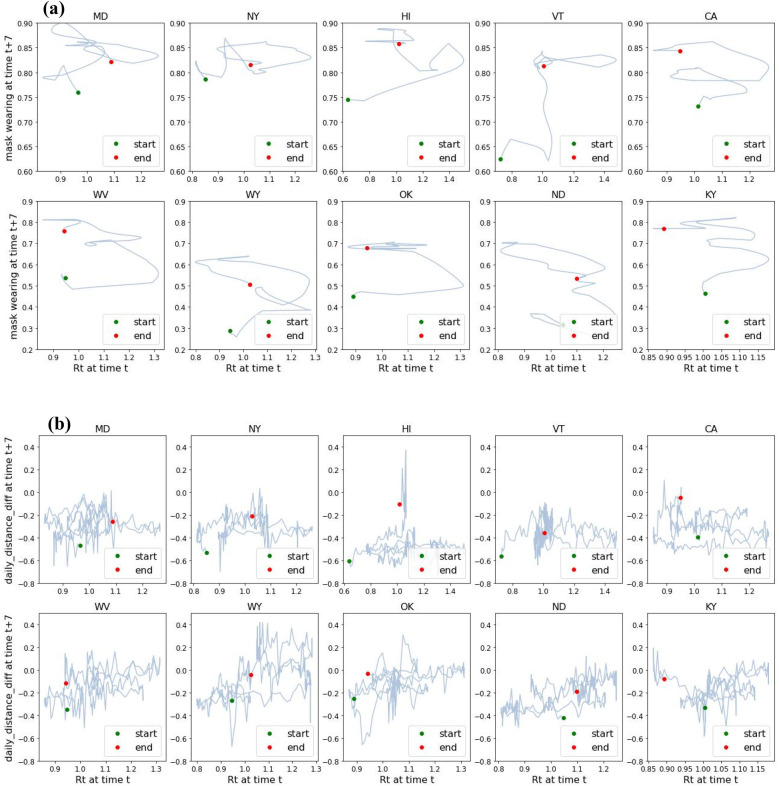
Daily behavior as a function of *R_t_*: **(a)** *R_t_* vs. proportion of mask wearing 7 days later, and **(b)** *R_t_* vs. social distancing 7 days later. A spiraling increase in the adoption of mask wearing can be observed in many cases as time progresses in **(a)**, whereas the pattern is more erratic in **(b)**. Figure panel titles are U.S. state abbreviations.

We investigated the use of Regional Psychologically Valid Agents, built upon the ACT-R cognitive architecture, to model two behaviors of non-pharmaceutical interventions of COVID-19. The R-PVA models performed better with mask wearing than with difference in social distance. At first glance, the two behaviors could be expected to have similar signatures. However, we note that while (increasing or decreasing) social distancing was a modification of a pre-existing construct, mask wearing was, for most of the population in the US, a novel behavior. Modifying an entrenched behavior is perhaps more prone to “relapse” than adopting a new behavior. Another possible dimension is that while mask wearing (including mask wearing in an ineffective way) is arguably compatible with most daily activities, increase in social distancing is harder to maintain in some situations, for instance birthday parties, which would induce people to sidestep the requirements for social distancing more often than for mask wearing. Figure 7 shows the evolution of *R_t_* _+_ _7_ as a function of *R_t_* seven days earlier that exhibits a signature temporal phenomenon observed in virtually all regions: a damped oscillation pattern around *R_t_* = 1. Figure 8a is a plot of *R_t_* vs. mask wearing 7 days later, and Figure 8b is a similar plot of the evolution of *R_t_* vs. the difference in social distance 7 days later. Compared to the corresponding plot of *R_t_* vs. mask wearing (Figure 8a), *R_t_* vs. social distancing (Figure 8b) showed a more erratic relationship to the combination of *R_t_* and time, indicating possible short-term relapses in social distancing. In addition, the upward spiral progression in Figure 8a suggests a self-efficacy component. The same pattern was not apparent for social distancing, suggesting that perhaps a different supplementary mechanism was at work. This was borne out by the results that self-efficacy contributed to better performance of R-PVA-mask-4, but not to the performance of R-PVA-distance-4.

Computational cognitive modeling is typically used in modeling individual cognition. In our research we have extended these modeling techniques to make time series predictions of decisions and behaviors that affect pandemic dynamics. Psychologically Valid Agents are needed for improved infectious disease control. They can help shape the course of human behavior response in more precise and less burdensome ways and aid in the development of new technologies for scenario modeling and mitigation efforts. This approach based on a unified theory of human cognition promises generalizability and deeper understanding of at-scale interactions between human behavior and pathogens and we recommend the approach for modeling the intricacies of the human mind as a foundation for future success in mitigating pandemics.

## Data Availability

The datasets will be available by request to the corresponding author, with the exception of the proprietary data from Unacast. Requests to access the datasets should be directed to Choh Man Teng, cmteng@ihmc.org; Unacast, https://www.unacast.com/post/the-unacast-social-distancing-scoreboard.

## Footnotes

^1^ https://www.pewresearch.org/politics/2022/03/03/the-changing-political-geography-of-covid-19-over-the-last-two-years/
