# Supplementary material for: Prediction of U.S. daily mask wearing and social distancing using psychologically valid agents during three waves of COVID-19
Source: Front Epidemiol. 2025 May 14;5:1532553. doi: 10.3389/fepid.2025.1532553 (PMC12116573; doi:10.3389/fepid.2025.1532553)
Supplement: Supplementary file 1 [file Datasheet1.pdf]

# *Supplementary Material*

## 1 Data

Tables S1 and S2 show the static and time-series data used in this study. The sources and types of data are as follows:

- CASOS: twitter data from CASOS group at Carnegie Mellon University collected by author Kathleen Carley (Uyheng et al., 2023)
- Unacast: geotracking data from Unacast that provides location tracking and map services using approximately 15 million U.S. smartphone GPS coordinates per day. These opt-in GPS data are categorized in terms of stops and travel events. The data were anonymized and aggregated at the county level: https://www.unacast.com/post/social-distancing-scoreboard
- John Hopkins: Johns Hopkins Coronavirus Resource Center (Johns Hopkins Coronavirus Resource Center, n.d.; Killeen et al., 2020)
- Gollwitzer: Data associated with a study on predictors of physical distancing (Gollwitzer et al., 2020)
- Ebert: regional Big 5 personality statistics (Ebert et al., 2022)
- covidestim.org: Yale School of Public Health (Chitwood et al., 2021)
- COVIDcast: Delphi COVID-19 Trends and Impact Survey (Reinhart et al., 2021)
- Stanford Cable TV News Analyzer (Hong et al., 2021)
- COVID States: survey (Gollwitzer et al., 2020)

**Table S1. Static variables**

| **Type** | **Variable** | **Description** | **Source** |
|---|---|---|---|
| Weather | PC1_weather | Derived weather temperature factor based on PCA | Johns Hopkins |
| | PC2_weather | Derived weather fall-winter precipitation factor based on PCA | Johns Hopkins |
| Race/ethnicity/gender | NHWA | Percentage of Non-Hispanic, White, living alone | Johns Hopkins |
| | NHBA | Percentage of Non-Hispanic, Black, living alone | Johns Hopkins |
| | NHIA | Percentage of Non-Hispanic, American Indian, living alone | Johns Hopkins |
| | TOM | Percentage of Two or more races | Johns Hopkins |
| | Gender ratio | Total male/total female population | Johns Hopkins |
| Age | Percentage age 0-17 yr | | Johns Hopkins |
| | Percentage age 65+ yr | | Johns Hopkins |
| Education | Percentage adults with less than a high school diploma | | Johns Hopkins |
| | Percentage adults with a bachelor's degree or higher | | Johns Hopkins |
| Economics | Median household income 2018 | | Johns Hopkins Johns Hopkins |
| | Unemployment rate 2018 | | Johns Hopkins |

| | | | |
|---|---|---|---|
| Media Diet | Fox News Lean | Proportion of people reporting watching Fox News minus proportion of people reporting watching CNN and MSNBC, 2019 | Gollwitzer |
| Politics | GOP Advantage 2016 | Percent Republican vote minus percent Democrat vote, 2016 | Gollwitzer |
| | PctTrump State 2016 | Percent Trump vote, 2016 | Gollwitzer |
| | Trump approval 2020 | Trump net approval (i.e., approval minus disapproval), -1 to 1 scale, February 2020 | Gollwitzer |
| | pol_gov_R | Whether the governor of a given state was a Republican | CASOS |
| Population | Population density per square mile | | Gollwitzer |
| Personality (Big 5) | Extraversion | Talkative, energetic, assertive, and outgoing | Ebert |
| | Openness | Willing to listen to multiple viewpoints or try new things. Those who are lower in openness tend to be averse to change and skeptical of new ideas. | Ebert |
| | Agreeableness | A tendency to get along well with others, even if one does not completely agree with their ideas or actions. | Ebert |
| | Conscientiousness | Organized, reliable, and ambitious | Ebert |
| | Emotional Stability | Ability to cope with stress, resist impulses, and adapt to change. People who score high on emotional stability tend to be calm, composed, and stress-resistant. | Ebert |

## Table S2. Timeseries and behavioral variables

| Type | Variable | Description | Source |
|---|---|---|---|
| Epidemiology | Rt | Effective reproductive number | covidestim.org |
| | CLI | Percentage of people with COVID-like illness | COVIDcast |
| | ILI | Percentage of people with influenza-like illness | COVIDcast |
| | whh_cmnty_cli | Percentage of people reporting illness in their local community, including their household | COVIDcast |
| | wnohh_cmnty_cli | Percentage of people reporting illness in their local community, not including their household | COVIDcast |
| | cdc_case_0_log10 | logged value of the number of COVID-19 cases | CASOS |
| Social Media | bot_prob_mean | Average bot probability of twitter accounts | CASOS |
| | lowcred_mean | Average number of links to low-credibility web domains in tweets | |
| | stance_pro_mean | Average number of tweets expressing a pro-mask stance | |
| | stance_con_mean | Average number of tweets expressing an anti-mask stance | |
| | hate_prob_mean | Average hate score of tweets | |

| | | | |
|---|---|---|---|
| | count_tweet | Number of tweets | |
| Mass Media | media_mask_protect | mentions of terms for "mask" with "protect" | Stanford Cable TV News Analyzer |
| | media_mask_restrict | mentions of terms for "mask" with "restrict" | |
| | media_mask_fear | mentions of terms for "mask" with "fear" | |
| | media_mask_positive | mentions of "mask" with positive terms | |
| | media_mask_negative | mentions of "mask" with negative terms | |
| Non-Pharmaceutical Interventions | mask wearing | | COVID States and COVIDcast |
| | daily distance difference | Change of average distance traveled from baseline | Unacast |

Table S3 shows the list of terms for each concept. Searches were performed for each of three channels (CNN, Fox News, MSNBC) using as search terms the conjunction of the disjunctions of the terms for the target concepts. Inflections were included for each term (e.g., "mask" would match both "mask" and "masks".) For example, the search term for "media_mask_protect" was the conjunction of three target concepts, "COVID", "mask" and "protect", with the following disjuncts for each concept.

> COVID: covid | coronavirus | corona virus | ...
> mask: mask | facemask | face shield | faceshield | PPE | ...
> protect: protection | guard | ward | shield | ...

The matched terms needed to occur within a period of 60 seconds of each other to be considered co-occurring. The value for each state was obtained by aggregating the values of the three TV channels in proportion to the percentage of population in that state watching each of the channels.

**Table S3. Terms for Mass Media Concepts**

| ***COVID*** | ***mask*** | ***protect*** | ***restrict*** | ***fear*** | ***positive*** | ***negative*** |
|---|---|---|---|---|---|---|
| covid | mask | protect | restrict | fear | effective | ineffective |
| coronavirus | facemask | defend | hamper | anxiety | efficient | inefficient |
| corona virus | face shield | safeguard | hinder | fearfulness | viable | useless |
| Wuhan flu | faceshield | shield | thwart | dread | useful | unhelpful |
| Wuhan pneumonia | PPE | guard | retard | panic | helpful | unacceptable |
| Wuhan virus | personal protection equipment | secure | impede | terror | protective | counter productive |
| Chinese virus | N95 | save | obstruct | fright | sufficient | counterproductive |
| SARS | N-95 | ward | limit | worry | adequate | pointless |
| severe acute respiratory syndrome | covering | buffer | confine | horror | strong | worthless |
| Omicron | | protection | block | trepidation | potent | unimportant |
| flu virus | | guard | repress | scare | acceptable | impractical |
| pandemic | | ward | inhibit | concern | worthwhile | impracticable |
| | | shield | restrain | dismay | important | infeasible |
| | | harbor | control | alarm | workable | unfeasible |
| | | safeguard | bridle | phobia | sensible | harmful |
| | | defense | constrain | nervousness | practical | unsafe |
| | | security | ban | apprehension | practicable | insufficient |
| | | buffer | compel | agitation | beneficial | inadequate |

| | | | | | | |
|---|---|---|---|---|---|---|
| | | | coerce | disquiet | harmless | weak |
| | | | force | qualm | preventive | inconclusive |
| | | | intimidate | discomposure | safe | unconvincing |
| | | | pressure | unease | suitable | unfounded |
| | | | bully | concernment | robust | invalid |
| | | | browbeat | uneasiness | powerful | unsound |
| | | | threaten | apprehension | significant | groundless |
| | | | restriction | disquietude | valid | wrong |
| | | | constraint | apprehensiveness | compelling | unsuitable |
| | | | prohibition | perturbation | convincing | authoritarian |
| | | | ban | discomfort | well founded | dictatorial |
| | | | coercion | doubt | well-founded | despotic |
| | | | | anxiousness | appropriate | tyrannical |
| | | | | stress | correct | tyrannic |
| | | | | distress | valuable | overbearing |
| | | | | consternation | invaluable | controlling |
| | | | | upset | vital | obtrusive |
| | | | | strain | critical | unnecessary |
| | | | | misgiving | essential | unworkable |
| | | | | vexation | necessary | nonviable |
| | | | | compunction | reasonable | unrealizable |
| | | | | fret | credible | unreasonable |
| | | | | afraid | creditable | irrational |
| | | | | scared | logical | nonrational |
| | | | | frightened | rational | illogical |
| | | | | terrified | justifiable | incoherent |
| | | | | fearful | well grounded | misleading |
| | | | | horrified | well-grounded | fallacious |
| | | | | alarmed | defensible | stupid |
| | | | | shocked | scientific | unscientific |
| | | | | worried | justified | preposterous |
| | | | | upset | substantiated | fatuous |
| | | | | aghast | actionable | nonsensical |
| | | | | apprehensive | feasible | nutty |
| | | | | appalled | realizable | loony |
| | | | | disturbed | doable | looney |
| | | | | horror-struck | believable | harebrained |
| | | | | horror struck | serviceable | daff |
| | | | | wary | advisable | daffy |
| | | | | perturbed | healthful | cockeyed |
| | | | | uneasy | healthy | half baked |
| | | | | disquieted | painless | half-baked |
| | | | | disconcerted | true | uncompelling |
| | | | | dismayed | prudent | half-witted |
| | | | | doubtful | good | unjustified |
| | | | | chagrined | great | unjustifiable |
| | | | | irritated | excellent | unwarranted |
| | | | | bothered | superior | unsupported |
| | | | | aggravated | better | unsubstantiated |
| | | | | mortified | best | implausible |
| | | | | nervous | outstanding | flimsy |
| | | | | distressed | terrific | nonvalid |
| | | | | | satisfactory | misguided |
| | | | | | decent | misled |

| | | | | | | |
|---|---|---|---|---|---|---|
| | | | | | commonsensical | unrealistic |
| | | | | | accurate | dubious |
| | | | | | legit | undoable |
| | | | | | legitimate | unbelievable |
| | | | | | | far-fetched |
| | | | | | | far fetched |
| | | | | | | outlandish |
| | | | | | | damaging |
| | | | | | | injurious |
| | | | | | | deleterious |
| | | | | | | adverse |
| | | | | | | detrimental |
| | | | | | | evil |
| | | | | | | hazardous |
| | | | | | | unhealthy |
| | | | | | | unhealthful |
| | | | | | | harsh |
| | | | | | | heavy-handed |
| | | | | | | heavy handed |
| | | | | | | bullying |
| | | | | | | false |
| | | | | | | incorrect |
| | | | | | | ridiculous |
| | | | | | | absurd |
| | | | | | | unsatisfactory |
| | | | | | | poor |
| | | | | | | inaccurate |
| | | | | | | terrible |
| | | | | | | lame |
| | | | | | | horrible |
| | | | | | | awful |
| | | | | | | disastrous |
| | | | | | | flawed |
| | | | | | | pathetic |
| | | | | | | dreadful |
| | | | | | | lousy |
| | | | | | | defective |
| | | | | | | unlawful |
| | | | | | | vile |
| | | | | | | rotten |
| | | | | | | illegal |
| | | | | | | reprehensible |

## 2 Correlations

**Figure S1. Correlation matrix for static, timeseries, and dependent behavior variable.**

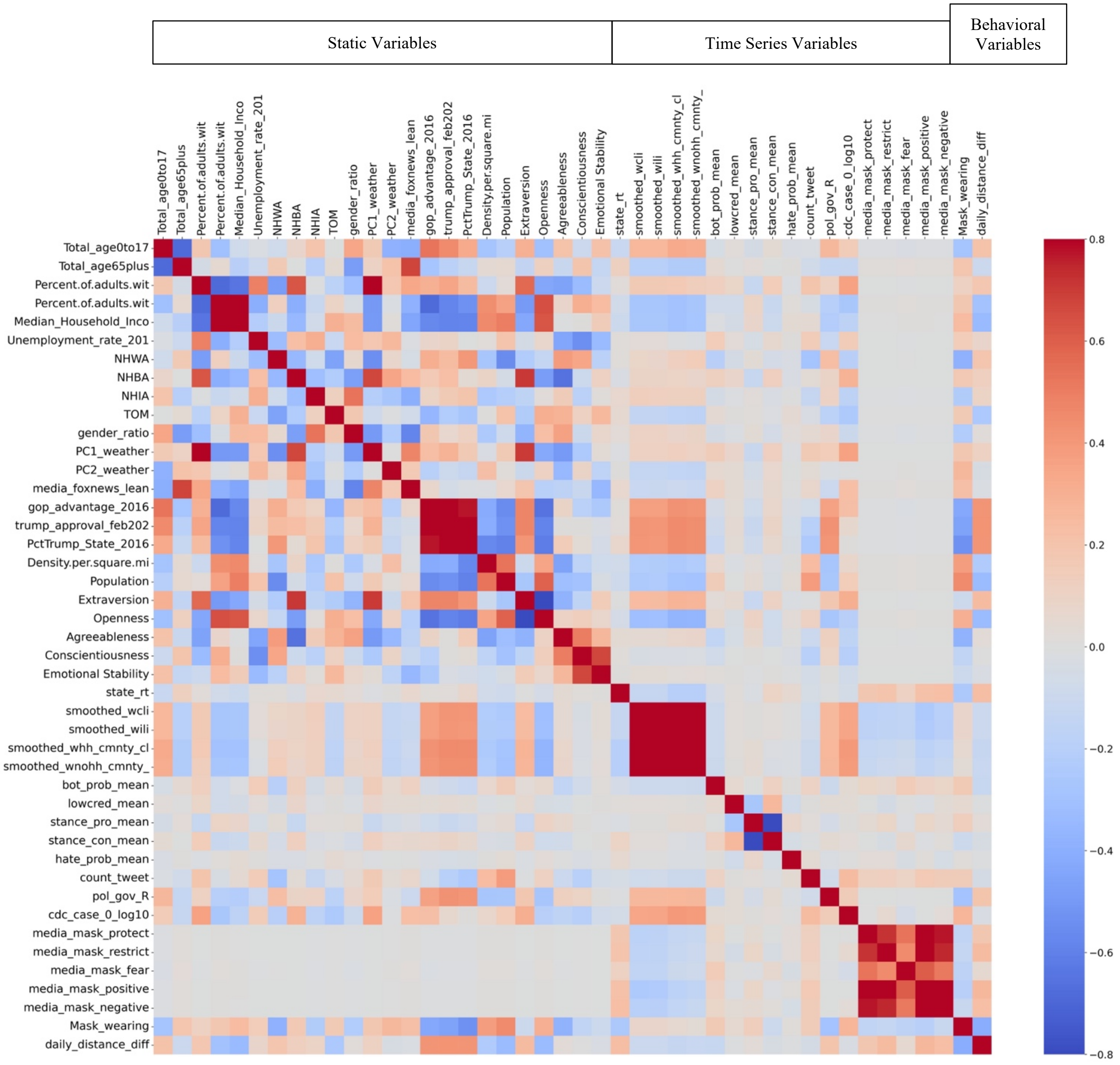


## 3 REFERENCES

Chitwood, M. H., Russi, M., Gunasekera, K., Havumaki, J., Pitzer, V. E., Salomon, J. A., Swartwood, N., Warren, J. L., Weinberger, D. M., Cohen, T., & Menzies, N. A. (2021). Reconstructing the course of the COVID-19 epidemic over 2020 for US states and counties:

results of a Bayesian evidence synthesis model. *medRxiv*, 2020.2006.2017.20133983. https://doi.org/10.1101/2020.06.17.20133983

Ebert, T., Gebauer, J. E., Brenner, T., Bleidorn, W., Gosling, S. D., Potter, J., & Rentfrow, P. J. (2022). Are Regional Differences in Psychological Characteristics and Their Correlates Robust? Applying Spatial-Analysis Techniques to Examine Regional Variation in Personality. *Perspectives on Psychological Science*, *17*(2), 407-441. https://doi.org/10.1177/1745691621998326

Gollwitzer, A., Martel, C., Brady, W. J., Pärnamets, P., Freedman, I. G., Knowles, E. D., & Van Bavel, J. J. (2020). Partisan differences in physical distancing are linked to health outcomes during the COVID-19 pandemic. *Nature Human Behaviour*, *4*(11), 1186-1197. https://doi.org/10.1038/s41562-020-00977-7

Hong, J., Crichton, W., Zhang, H., Fu, D. Y., Ritchie, J., Barenholtz, J., Hannel, B., Yao, X., Murray, M., Moriba, G., Agrawala, M., & Fatahalian, K. (2021). *Analysis of Faces in a Decade of US Cable TV News* Proceedings of the 27th ACM SIGKDD Conference on Knowledge Discovery & Data Mining, Virtual Event, Singapore. https://doi.org/10.1145/3447548.3467134

Johns Hopkins Coronavirus Resource Center. (n.d.). (https://coronavirus.jhu.edu/about/how-to-use-our-data

Killeen, B. D., Wu, J. Y., Shah, K., Zapaishchykova, A., Nikutta, P., Tamhane, A., Chakraborty, S., Wei, J., Gao, T., Thies, M., & Unberath, M. (2020). A County-level Dataset for Informing the United States' Response to COVID-19. arXiv:2004.00756. Retrieved April 01, 2020, from https://ui.adsabs.harvard.edu/abs/2020arXiv200400756K

Reinhart, A., Brooks, L., Jahja, M., Rumack, A., Tang, J., Agrawal, S., Al Saeed, W., Arnold, T., Basu, A., Bien, J., Cabrera, Á. A., Chin, A., Chua, E. J., Clark, B., Colquhoun, S., DeFries, N., Farrow, D. C., Forlizzi, J., Grabman, J.,…Tibshirani, R. J. (2021). An open repository of real-time COVID-19 indicators. *Proceedings of the National Academy of Sciences*, *118*(51), e2111452118. https://doi.org/doi:10.1073/pnas.2111452118

Uyheng, J., Robertson, D. C., & Carley, K. M. (2023). Bridging online and offline dynamics of the face mask infodemic. *BMC Digital Health*, *1*(1), 27. https://doi.org/10.1186/s44247-023-00026-z
